# Design of Cinnamaldehyde- and Gentamicin-Loaded Double-Layer Corneal Nanofiber Patches with Antibiofilm and Antimicrobial Effects

**DOI:** 10.1021/acsomega.3c00914

**Published:** 2023-07-26

**Authors:** Sumeyye Cesur, Elif Ilhan, Tufan Arslan Tut, Elif Kaya, Basak Dalbayrak, Gulgun Bosgelmez-Tinaz, Elif Damla Arısan, Oguzhan Gunduz, Ewa Kijeńska-Gawrońska

**Affiliations:** † Center for Nanotechnology & Biomaterials Application and Research (NBUAM), Marmara University, Istanbul 34722, Turkey; ‡ Department of Metallurgical and Materials Engineering, Faculty of Technology, Marmara University, Istanbul 34722, Turkey; § Department of Basic Pharmaceutical Sciences, Faculty of Pharmacy, Marmara University, Istanbul 34668, Turkey; ∥ Department of Biotechnology, Institute of Biotechnology, Gebze Technical University, Gebze 41400, Kocaeli, Turkey; ⊥ Centre for Advanced Materials and Technologies CEZAMAT, Warsaw University of Technology, 02-822 Warsaw, Poland; # Faculty of Materials Science and Engineering, Warsaw University of Technology, 02-507 Warsaw, Poland

## Abstract

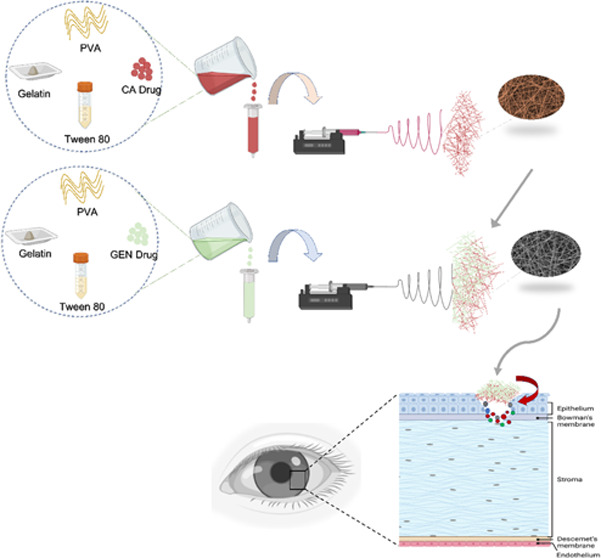

In this study, two-layer poly(vinyl alcohol)/gelatin (PVA/GEL) nanofiber patches containing cinnamaldehyde (CA) in the first layer and gentamicin (GEN) in the second layer were produced by the electrospinning method. The morphology, chemical structures, and thermal temperatures of the produced pure (PVA/GEL), CA-loaded (PVA/GEL/CA), GEN-loaded (PVA/GEL/GEN), and combined drug-loaded (PVA/GEL/CA/GEN) nanofiber patches were determined by scanning electron microscopy (SEM), Fourier transform infrared spectroscopy, and differential scanning calorimetry, respectively. Their mechanical properties, swelling and degradation behavior, and drug release kinetics were investigated. SEM images showed that both drug-free and drug-loaded nanofiber patches possess smooth and monodisperse structures, and nanofiber size increase occurred as the amount of drug increased. The tensile test results showed that the mechanical strength decreased as the drug was loaded. According to the drug release results, CA release ended at the 96th hour, while GEN release continued until the 264th hour. The antibacterial and antibiofilm activities of PVA/GEL, PVA/GEL/CA, PVA/GEL/GEN, and PVA/GEL/CA/GEN nanofiber patches against *Pseudomonas aeruginosa* and *Staphylococcus aureus* were evaluated. Results showed that PVA/GEL/GEN and PVA/GEL/CA/GEN nanofiber patches have excellent antibacterial and antibiofilm activities. Moreover, all materials were biocompatible, with no cytotoxic effects in the mammalian cell model for 8 days. PVA/GEL/GEN nanofiber patches were the most promising material for a high cell survival ratio, which was confirmed by SEM images. This research aims to develop an alternative method to stop and treat the rapid progression of bacterial keratitis.

## 1 Introduction

Bacterial keratitis, also known as corneal ulcer or microbial keratitis, sustains one of the main causes of corneal blindness in the world.^1^ This common ocular infection can be caused by fungi, bacteria, viruses, and parasites;^2^ its treatment demands broad-spectrum and intensive antibiotic and drug therapy.^3^ A disease that can occur in less than 24 h in the presence of invasive pathogens such as *Pseudomonas aeruginosa* and *Staphylococcus aureus*, it is one of the most threatening ocular infections due to its high incidence and complications.^4^ Although the basis of bacterial keratitis treatment requires intensive use of antibiotics, the effectiveness of therapy decreases due to the rise of antibiotic resistance in bacteria.^5,6^ Moreover, complications such as corneal melting, perforation, and endophthalmitis may occur despite intensive antibiotic therapy and necessitate further operational treatment, including tectonic or therapeutic keratoplasty.^3^

Studies have shown that approximately 40–80% of bacterial cells can form biofilms.^7^ More than 90% of chronic wounds or ulcers are caused by biofilms. Biofilms are communities of microorganisms primarily composed of polysaccharides, secreted proteins, and extracellular DNA held together by a self-generated polymer matrix (EPS) that allows them to adhere to any surface or each other.^8,9^ In biofilm formation, first, planktonic bacteria attach to a surface and become more resistant to antibiotics, antiseptics, and disinfectants within 6–12 h. Moreover, bacteria within the biofilm can be up to 1000 times more resistant to antibiotics.^10^ The main reason for this high resistance is the inability of effective concentrations of antibiotics to pass through the EPS matrix and reach the bacteria in the biofilm. In addition, the metabolic rate of the bacteria at the base of the biofilm is very low, and the low metabolic rate significantly reduces the effects of antibiotics and increases antibiotic resistance.^11^ Several animal experiments have confirmed that biofilm formation in wounds delays wound healing.^12−15^ Therefore, new approaches for wound healing and prevention of biofilm formation are needed in the treatment of biofilm-associated infections.

Natural plant products, primarily phytochemicals, have historically been accepted as an alternative to antibacterial agents. They have the advantages of being inexpensive, having low levels of cytotoxicity, being less susceptible to evolve resistance to antibiotics, and being an abundant source.^16,17^ Cinnamaldehyde, an α,β-unsaturated aromatic aldehyde, is an important constituent of cinnamon essential oil.^18^ It has been reported by previous studies that cinnamaldehyde has antibacterial activity and is effective against bacterial biofilms formed by Gram-positive and Gram-negative bacteria such as *P. aeruginosa* and *S. aureus.*(19,20) Gentamicin is a broad-spectrum antibiotic that is used in the treatment of various contagions evoked by predominantly Gram-negative bacteria, including *P. aeruginosa*, *Escherichia coli*, *Enterobacter aerogenes*, and the Gram-positive *S. aureus*. Gentamicin is on the World Health Organization’s Essential Medicines list due to its low toxicity.^21^ Many studies provide evidence of the effectiveness of gentamicin on bacterial keratitis.^22,23^

Electrospinning is a simple, versatile nanofiber production method^24^ that allows the production of biomimetic scaffolds from natural and synthetic polymers, consisting of a large network of interconnected fibers and pores,^25^ allowing the proficient exchange of nutrients and metabolic wastes, thanks to its high porosity.^26^ In this study, biocompatible and biodegradable poly(vinyl alcohol) (PVA) has been reported in the literature to promote oxygen permeability, which can be a crucial feature for corneal tissue engineering field.^27,28^ On the other hand, due to its excellent transparency, gelatin (GEL) has been used in ocular tissue engineering.^29^

In this study, PVA/GEL nanofibers enriched with cinnamaldehyde and gentamicin were produced with an electrospinning method aiming to develop an alternative method for bacterial keratitis treatment by designing nanofibrous layers that inhibit biofilm formation and could serve as a corneal patch. The effects on the physical and chemical properties, drug release profiles, antimicrobial and antibiofilm features, and in vitro cell viability of the gentamicin-and cinnamaldehyde-loaded nanofiber patches were investigated.

## 2 Materials and Methods

### 2.1 Materials

Gentamicin (GEN, potency: 50–60 mg/mL) and cinnamaldehyde (CA) were purchased from Sigma-Aldrich, Darmstadt, Germany. Poly(vinyl alcohol) (PVA), *M*_w_: 89,000–98,000 (99+% hydrolyzed), gelatin from bovine skin (GEL, gel strength ∼225 g bloom, Type B), glutaraldehyde solution (GA, 50% wt, *M*_w_: 100.12 g/mol), Tween 80, phosphate buffer saline (PBS, pH = 7.4), Mueller–Hinton Agar, and Luria Bertani (LB) broth were bought from Sigma-Aldrich (St. Louis, MO). Crystal violet was obtained from Merck. Mouse embryonic fibroblast (MEF) cells (SCRC-1040, ATCC), DMEM high glucose with 4.5 g/L d-glucose, l-glutamine, and sodium pyruvate (NutriCulture, Eco Biotech), phosphate buffer saline (PBS) (Eco Biotech), fetal bovine serum (Gibco), 1% penicillin–streptomycin (PAN Biotech), DiOC6 (3,3′-dihexyloxacarbocyanine iodide) (Thermo Fisher), and propidium iodine (Sigma-Aldrich) were used in the study.

### 2.2 Preparation of Electrospinning Solutions

Solutions of different concentrations were prepared, as demonstrated in Table 1. First, 13 wt % PVA was dissolved in a magnetic stirrer (WiseStir, MSH-20 Germany) at 90 °C with stirring for about 1 h. After dissolving, 3 wt % Tween 80 was added to this solution and stirred for an additional 15 min. Then, 0.5 wt % GEL was added, and the solution was stirred at 60 °C for another 15 min. Finally, 0.5 wt % GEN and 2.6 wt % CA were added separately into the solution and mixed for 30 min.

**Table 1:** Contents of the Solutions Used to Produce All Types of Nanofiber Patches

nanofiber patches	PVA content (wt %)	GEL content (wt %)	Tween 80 (wt %)	drug amount (wt %)
PVA/GEL	13	0.5	3	
PVA/GEL/CA	13	0.5	3	2.6
PVA/GEL/GEN	13	0.5	3	0.5
PVA/GEL/CA/GEN	13	0.5	3	2.6/0.5

### 2.3 Fabrication of Nanofiber Patches

**Figure 1:**
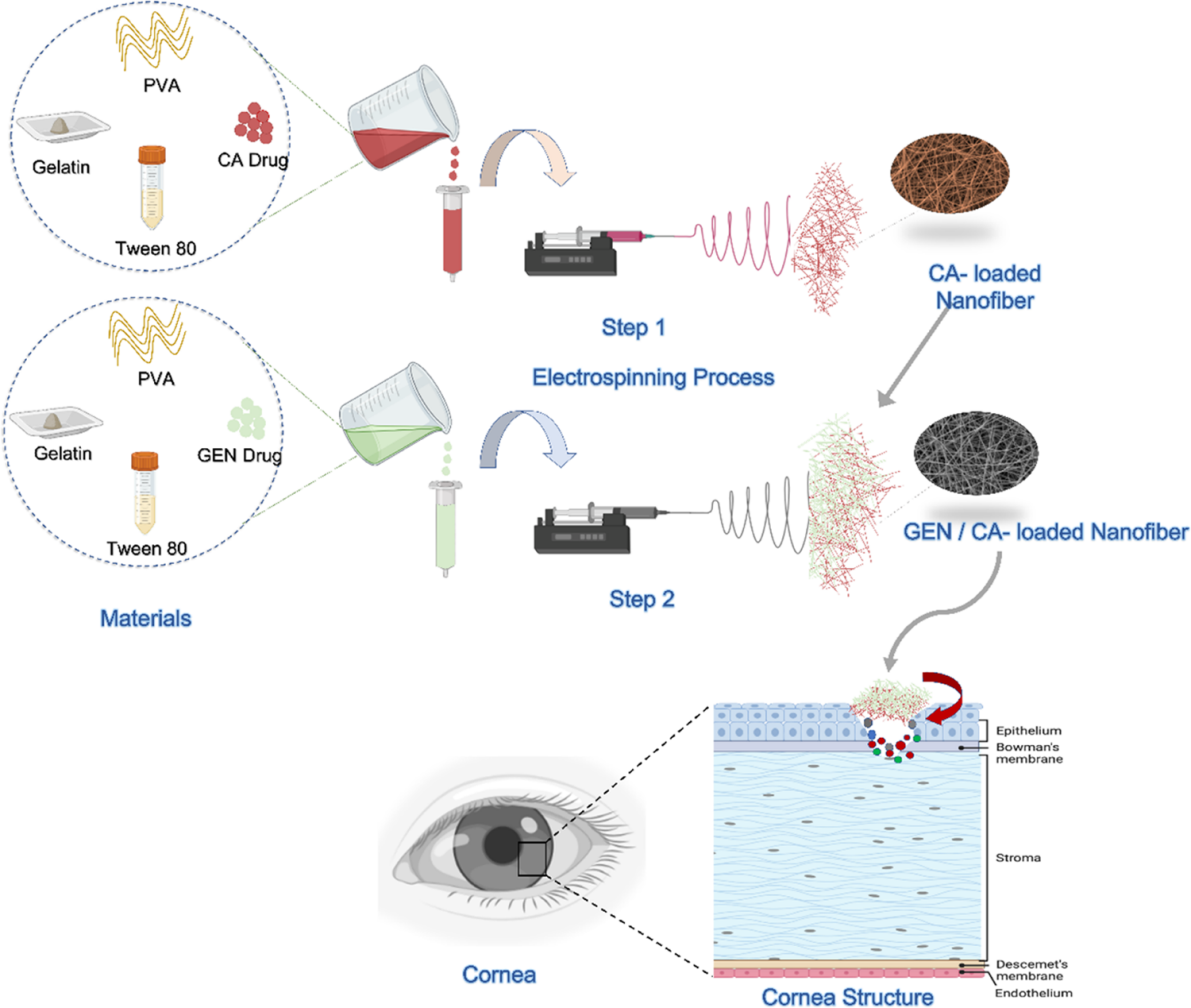
Schematic representation of the electrospinning process and application for the treatment of bacterial keratitis. Created with BioRender.com.

Pure and drug-loaded nanofiber patches were produced by adjusting the flow rate, voltage, and working distance (the distance between the needle tip and the collector) by the electrospinning method. For this, a laboratory-scale electrospinning device (NS24, Inovenso Co., Turkey), syringe pump (NE-300, New Era Pump Inc.), and power supply were used. The applied voltage was set to 25 kV, the flow rate was 0.5 mL/h, and the working distance was 12 cm. Pure and GEN- and CA-loaded nanofiber patches were produced separately for 7 h each. Solutions containing GEN and CA were produced in a single nanofiber patch as separate layers a total 7 h. In the first layer, GEN was electrospun for 3 h and 30 min and then CA on top for 3 h and 30 min (Figure 1).

### 2.4 Crosslinking of Nanofiber Patches

Crosslinking of nanofiber patches was performed using 25% GA vapor. The nanofiber patches were placed in a desiccator over the glutaraldehyde solution and incubated in an oven at 40 °C for 1 h.

### 2.5 Observations of the Morphology of the Fibers

The morphology of the nanofiber patches was studied using an EVO 40 LS 10 Zeiss scanning electron microscope (SEM). The surface of the samples was spray-coated with gold and palladium for 120 s with a spray coating machine (Quorum SC7620) prior to SEM observation. According to the obtained SEM results, fiber distribution and diameter were measured using image software (Olympus AnalySIS).

### 2.6 Evaluation of the Functional Groups of Nanofibrous Patches

Fourier transform infrared spectroscopy (FTIR) was used to determine the chemical bond structures and functional groups of nanofiber patches. A Jasco FT/IR-4700 machine was used for these measurements. Spectra were recorded at a resolution of 4 cm^–1^ at room temperature (23 °C) at 32 scan rates from 400 to 4000 cm^–1^.

### 2.7 Characterization of the Thermal Properties of the Fibers

Differential scanning calorimetry (DSC) (Shimadzu, Japan) was used for the characterization of the thermal properties of the material. For all nanofiber patches, the heating rate was chosen as 10 °C min^–1^, and the temperature ranges were set between 25 and 300 °C.

### 2.8 Tensile Tests of Nanofiber Patches

A tensile testing device was used to determine and interpret the mechanical properties of the nanofiber patches (Shimadzu Corporation, EZ-LX, Kyoto, Japan). For this, the nanofiber patches were cut into a rectangle of size of 10 mm × 50 mm. Before starting the test, the thickness of the nanofiber patches was measured using a digital micrometer (Mitutoyo MTI Co.). The test speed was set to 5 mm/min, and a force of 0.1 N was applied during the test. The measurement was repeated 3 times for each group.

### 2.9 Swelling and Degradation

Swelling and degradation tests were applied to nanofiber patches. Phosphate-buffered saline (PBS) solution with a pH value of 7.4, mimicking human plasma, was used for the swelling and degradation test. The initial weights (*W*_0_) of the samples were recorded before starting the test. For the swelling test, nanofiber patch pieces with equal weights were dipped in PBS solution in 1 mL Eppendorf tubes and kept in a thermal shaker (BIOSAN-TS 100, Riga, Latvia) at 37 °C. The nanofiber patches’ initial weights were noted. Daily measurements were made of the samples’ wet weights. The swelling value (*S*) was calculated using eq 1(30)
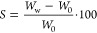
1The initial weights (*W*_0_) of nanofiber patches were weighed. Weighed nanofiber patch pieces were kept in PBS solution in 1 mL Eppendorf tubes for 24 h in a thermal shaker at 37 °C for the degradation test. After this step, PBS was removed from the samples, and wet nanofiber patches were kept in an incubator at 37 °C for 24 h with the lids of Eppendorf tubes open. The samples’ dry weights (*W*_t_) were weighed, and the results were recorded. As a result of the measurements taken every other day, the degradation value (*D*) was calculated according to eq 2(31)
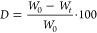
2

### 2.10 In Vitro Drug Release

In vitro release testing of drug-loaded nanofiber patches was performed in a pH 7.4 PBS medium. Each of the drug-loaded nanofiber patches weighing 5 mg was transferred to an Eppendorf tube containing 1 mL of PBS. During the test, Eppendorf tubes were kept at 37 °C, 380 rpm in a thermal shaker (BIOSAN-TS 100, Riga, Latvia). At the specified time periods, the drug-loaded nanofibers in the tubes were removed from 1 mL of PBS, and the removed 1 mL PBS medium was tested in the UV spectrophotometer (Shimadzu UV-3600, Japan) between the wavelength range of 190 and 500 nm. After measuring the drug release at certain time points, 1 mL of fresh PBS was returned to each tube. The amount of the drugs was calculated based on the calibration curves prepared for different concentrations of cinnamaldehyde and gentamicin, respectively, and both absorption and spectra graphs are presented in Figure S1.

### 2.11 Drug Release Kinetics

Mathematical models were used to examine, interpret, and compare the release kinetics of drugs. Drug release kinetics of nanofiber patches was determined by using zero-order (3), first-order (4), Korsmeyer–Peppas (5), Higuchi (6), and Hixson–Crowell (7) mathematical models, respectively, as follows:^32^
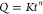
3
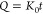
4
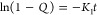
5
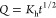
6
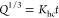
7The fractional amount of drug released at time *t* is symbolized by *Q*. In the above equations, the representations of *K*_0_, *K*_1_, *K*, *K*_h_, and *K*_hc_ are the kinetic constants of the zero-order, first-order, Korsmeyer–Peppas, Higuchi, and Hixson–Crowell models, respectively. “*n*,” the diffusion exponent, is indicative of the drug release mechanism.

### 2.12 In Vitro Antibacterial and Antibiofilm Analyzes of PVA/GEL/CA, PVA/GEL/GEN, and PVA/GEL/CA/GEN Nanofibers

The antibacterial activities of PVA/GEL, PVA/GEL/CA, PVA/GEL/GEN, and PVA/GEL/CA/GEN nanofiber patches against *S. aureus* ATCC 25923 and *P. aeruginosa* 27853 were tested using the disk diffusion method, as described by the Clinical Laboratory Standards Institute (CLSI). Overnight cultures of *S. aureus* ATCC 25923 and *P. aeruginosa* 27,853 were spread onto Mueller–Hinton Agar plates. PVA/GEL, PVA/GEL/CA, PVA/GEL/GEN, and PVA/GEL/CA/GEN nanofiber patches cut into 6 mm disks were placed on the agar plates and incubated at 37 °C for 24 h. Inhibition zones around the disks were determined by measuring the zone diameters with a ruler.

The antibiofilm capacity of PVA/GEL/CA, PVA/GEL/GEN, and PVA/GEL/CA/GEN nanofiber patches were analyzed by crystal violet (CV) staining.^33^ The overnight cultures of *P. aeruginosa* PAO1 and *S. aureus* ATCC 25923 were diluted to an optical density (OD_600_) of 0.05. One milliliter of diluted culture was transferred to polystyrene tubes and incubated at 37 °C with PVA/GEL/CA, PVA/GEL/GEN, and PVA/GEL/CA/GEN nanofiber patches. After 24 h, nonadherent cells were removed. After rinsing tubes with distilled water, the biofilms were dyed with 0.4% CV solution. For the quantification of biofilms, the biofilm-associated CV was solubilized with 95% ethanol, and the absorbance was measured at 595 nm using a microplate reader.

Biofilm inhibition was calculated by
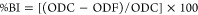
8%BI is the percentage of biofilm inhibition, ODC is the 595 nm absorbance value of control (without nanofiber patches), and ODF is the 595 nm absorbance value of the sample with nanofiber patches.

### 2.13 Cell Culture Test

#### 2.13.1 Cell Viability

Nanofiber patches were sterilized with UV under the laminar flow cabinet (Safe 2020, Thermo Scientific), and the nanofiber patches were put onto the 96-well plate for cell viability (MTT (3-(4,5-dimethylthiazol-2-yl)–2,5-diphenyltetrazolium bromide) assay and fluorescence imaging) and SEM imaging.

Mouse embryonic fibroblast (MEF) cells (SCRC-1040, ATCC) were incubated for the nanofiber patches’ cell viability tests with DMEM high glucose with 4.5 g/L d-glucose and l-glutamine, and sodium pyruvate (NutriCulture, Eco Biotech) medium was used with 10% fetal bovine serum (Gibco) and 1% penicillin–streptomycin (PAN Biotech). The cells were collected and counted after they reached 80% confluency. The appropriate number of cells (2.5 × 10^4^ cells/mL) were seeded onto the nanofiber patches, and the nanofiber patches with cells were incubated at 37 °C with 5% CO_2_ (Thermo). The cell seeding procedure was repeated 3 times to obtain 8-day, 3-day, and 1-day incubation time, and the experiments were repeated 3 times for statistical analysis.

10 μL of MTT reagent was applied after the incubation times were completed, and the cells were incubated for 4 more hours to obtain formazan crystals. Then, the existing medium was removed with dimethyl sulfoxide (DMSO) to solve crystal formations. The photometric analysis was acquired by Varioskan plate reader (Thermo Scientific) at 570 nm. The material control was used to calculate the cellular viability depending on the time. Two-way ANOVA and Tukey’s tests were applied for statistical analysis with GraphPad Prism 9.

Fluorescence images were taken with ZOE fluorescent cell imager (BIORAD) under the appropriate wavelengths, using 4 nM DiOC_6_ (3,3′-dihexyloxacarbocyanine Iodide) (Thermo Fisher) and 0.5 mg/mL PI (propidium iodine) (Sigma-Aldrich) to identify the living and the dead cells, respectively. After fluorescence dyes were applied to the cells with serum- and antibiotic-free DMEM, the cells were incubated for 15 min at the same conditions with growth.

#### 2.13.2 Scanning Electron Microscopy Images

The nanofiber patch preparation was achieved in the same procedure as cellular viability assays; after cellular incubation, the existing medium was discarded, and the nanofiber patches were washed with PBS (Eco Biotech) carefully. Then, the nanofiber patches were treated with a fixation solution (acetic acid: methanol, 1:3) for 15 min, and further, the nanofiber patches—cell constructs—were evaluated under SEM, (EVO LS 10, Zeiss).

### 2.14 Statistical Analysis

All experiments were carried out at least in triplicate, and data are expressed as mean ± standard deviation (SD). The results were evaluated statistically by means of post-hoc one-way ANOVA with a Tukey–Kramer pair-wise comparison test, and a value of *p* ≤ 0.05 was considered statistically significant, and additional significance was indicated with **p < 0.01 and ***p < 0.001. All statistical analyses were calculated using GraphPad Prism version 9.2.0 for Mac OS X (GraphPad Software, La Jolla, California).

## 3 Results and Discussion

### 3.1 Morphology of the Nanofibrous Patches

**Figure 2:**
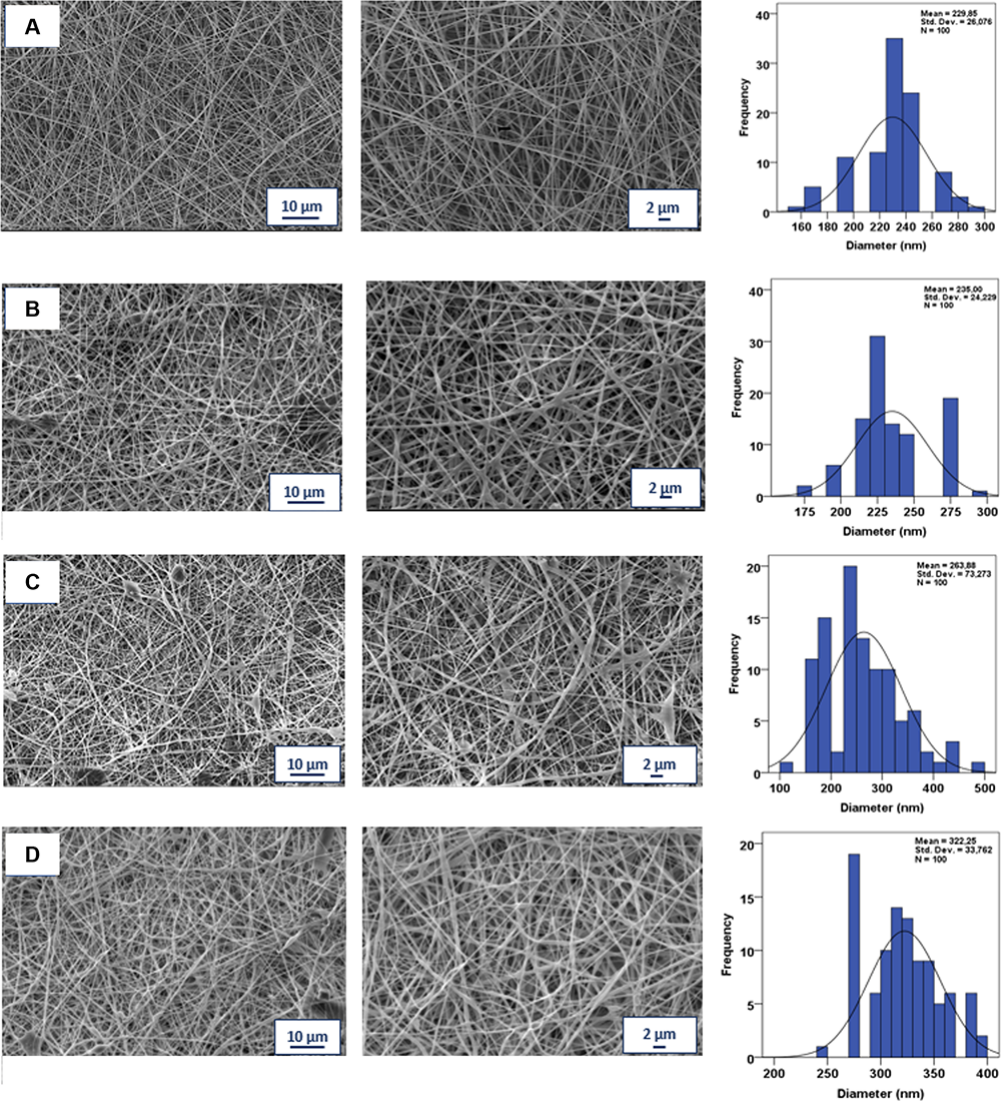
SEM images of the (A) PVA/GEL, (B) PVA/GEL/GEN, (C) PVA/GEL/CA, and (D) PVA/ GEL/CA/GEN nanofiber patches and their fiber diameter distributions.

The surface morphology of the nanofiber patches was investigated with SEM. Based on the SEM images of the nanofibers, diameter measurements were made with 100 random measurements for each sample, and diameter distribution graphs were drawn. Figure 2 represents SEM images and diameter distribution histograms of nanofiber patches. Non-drug and drug-loaded nanofiber patches showed highly uniform and smooth surface morphologies without any visible beads. As shown in Figure 2, PVA/GEL, PVA/GEL/GEN, PVA/GEL/CA, and PVA/ GEL/CA/GEN nanofiber patches were produced with mean diameters of 229 ± 26, 235 ± 24, 263 ± 73, and 322 ± 34 nm, respectively. It was observed that the diameter of the nanofiber patches increased with the loading of the drug. Other studies also support that the average diameter increases when the drug is loaded into the pure PVA nanofiber patches.^34^ Especially, a sharp increase was seen in the diameter of the PVA/GEL/CA/GEN nanofiber patches loaded with both types of drugs. Also, there are some studies that show that the diameter of the pure nanofiber patches increases with the loading of the GEN drug. In the study of Kimna et al., it was observed that the fiber diameter increased with the loading of the GEN drug into the pure zein fiber.^35^ In the present study, it was observed that drug loading to PVA/GEL/CA, PVA/GEL/GEN, and PVA/GEL/CA/GEN nanofiber patches was performed effectively. Thus, drug crystals and aggregates were not observed in the surface morphology of the nanofiber patches.

### 3.2 Chemical Properties of the Nanofibrous Patches

**Figure 3:**
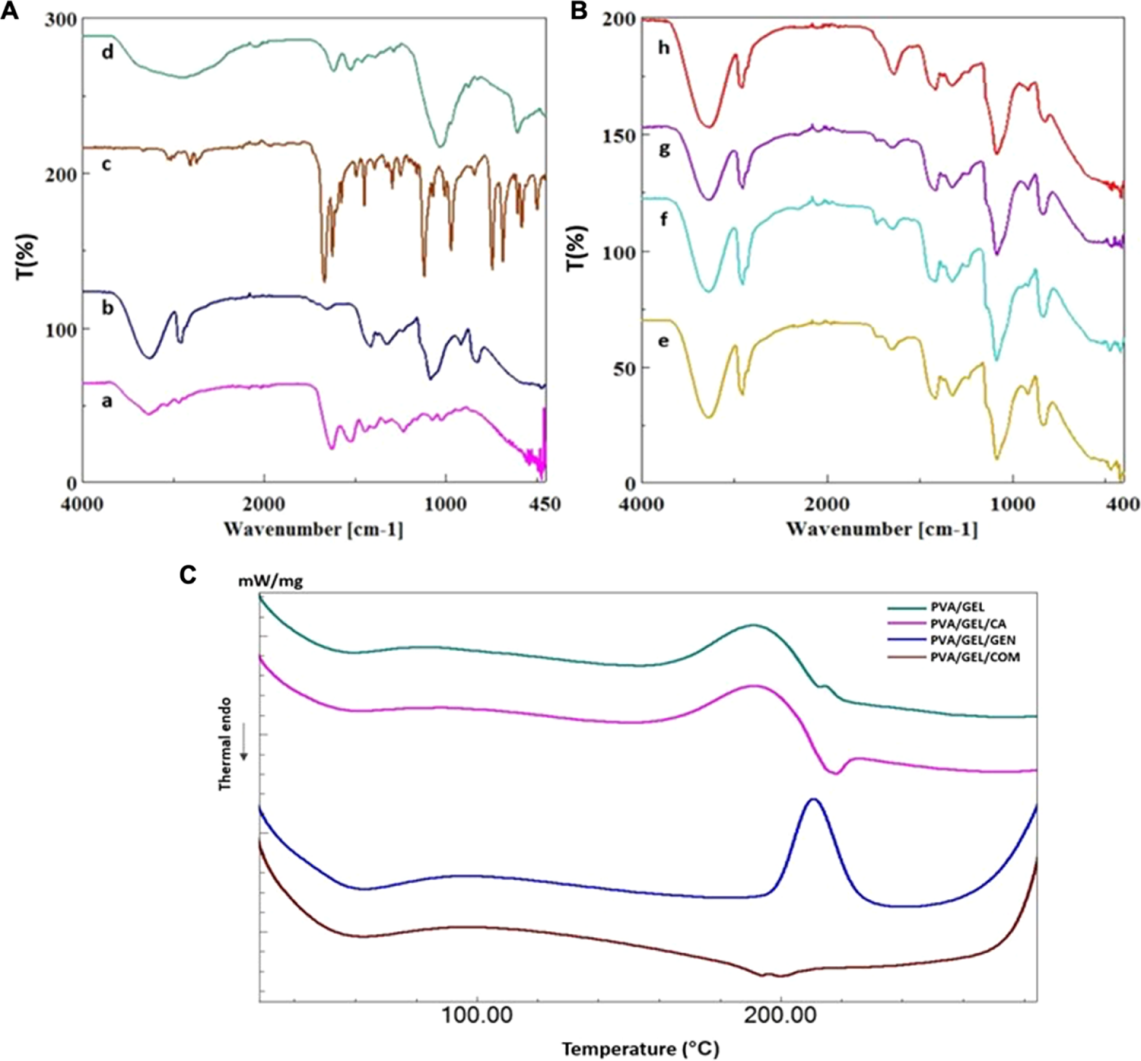
FTIR spectra of (A,a) pure GEL, (A,b) pure PVA, (A,c) CA, (A,d) GEN, (B,e) PVA/GEL, (B,f) PVA/GEL/CA, (B,g) PVA/GEL/GEN, and (B,h) PVA/GEL/CA/GEN nanofiber patches. (C) DSC thermograms of PVA/GEL, PVA/GEL/CA, PVA/GEL/GEN, and PVA/GEL/CA/GEN nanofiber patches.

FTIR analysis was used to determine chemical structures and functional groups of produced nanofiber patches. In Figure 3A,a, the peaks of pure GEL are seen. The peak, which is seen at ∼3277 cm^–1^, corresponds to absorption bands N–H stretching, and the peak at ∼2933 cm^–1^ indicates the C–H stretching. N–H bending is seen at ∼1525 cm^–1^. The peak at ∼1626 cm^–1^ indicates the C–O stretching.^36^ In Figure 3A,b, absorption peaks of pure PVA are shown. The peak at ∼3268 cm^–1^ is related to O–H stretching, which confirms the intramolecular and intermolecular hydrogen bonds.^21^ The peak at ∼2910 cm^–1^ corresponds to the C–H stretching of the alkyl groups. C=O and C–O stretching of the acetate groups are visible on the peak at ∼1646 cm^–1^. The peak at ∼1415 cm^–1^ indicates the C–H bending vibration of C–H_2_. The absorption peak at ∼1085 cm^–1^ corresponds to the CO group.^37^ CO stretching is observed at the peak at ∼ 831 cm^–1^. For CA, the absorption peaks are seen in Figure 3A,c. C=O was observed at the peak at ∼1670 cm^–1^ due to the aldehyde group in its molecular structure. The peak at ∼1616 cm^–1^ is connected to the aromatic benzene ring. Figure 3A,d shows the absorption peaks of GEN. C–H group arising from alkyl stretching vibrations is seen at ∼2883 cm^–1^. The N–H group sourced from the amide bending vibrations is visible at ∼1617 cm^–1^ [2]. The peak representing C–H stretching vibration is seen at ∼1525 cm^–1^. The peak indicating the S–O stretch was observed at ∼1031 cm^–1^.^38^ It was observed that all of the produced nanofiber patches presented in Figure 3B,e–h gave peaks at the same wavelength of ∼2910, ∼1415, and ∼1085 cm^–1^ as the pure PVA, as shown in Figure 3A,b. When the peak of PVA/GEL observed in Figure 3B,e at ∼831 cm^–1^ was compared to the peaks of the drug-loaded nanofiber patches, the transmittance was visibly lower.

### 3.3 Thermal Properties of the Nanofibrous Patches

DSC was used to determine the characteristic thermal features of the nanofiber patches, such as melting temperature (*T*_m_), glass-transition temperature (*T*_g_), phase changes, and heat capacity (*C*_p_).^39^ In Figure 3C, DSC thermograms of PVA/GEL, PVA/GEL/CA, PVA/GEL/GEN, and PVA/GEL/CA/GEN nanofiber patches were demonstrated. Since PVA has the highest concentration in all of the produced nanofiber patches, it was determined that the peaks observed as a result of the DSC analysis also belong to PVA. For the PVA/GEL nanofiber patches, the melting temperature of PVA was observed at 212.84 °C. The peak detected at 190.98 °C for the PVA/GEL nanofiber patches represents the endothermic curve. The glass-transition temperature of PVA is in the range of 50–60 °C.^36^ For PVA/GEL, PVA/GEL/CA, PVA/GEL/GEN, and PVA/GEL/CA/GEN, the glass-transition temperatures were observed at 59.42, 61.07, 63.13, and 62.87 °C, respectively. There was a slight shift in glass-transition temperatures with the addition of CA and GEN drugs to the non-drug-containing PVA/GEL nanofiber patches. When the melting point temperatures for PVA/GEL, PVA/GEL/CA, PVA/GEL/GEN, and PVA/GEL/CA/GEN were compared, they were observed as 214.53, 218.09, 210.76, and 199.75 °C. In addition, it was observed that the addition of CA and GEN drugs to PVA/GEL nanofiber patches caused small shifts in melting point temperatures.

### 3.4 Mechanical Properties of the Nanofiber Patches

**Figure 4:**
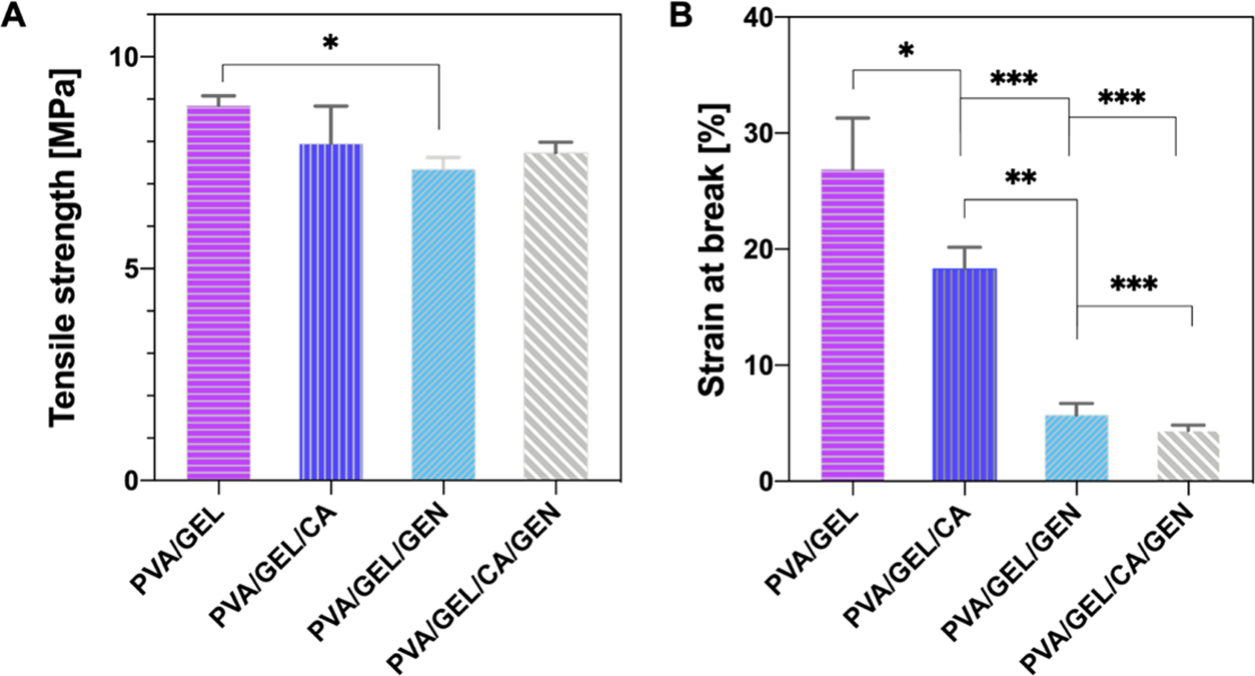
Mechanical test results of nanofibers patches: (A) tensile strength and (B) strain at break.

The mechanical properties of nanofiber patches vary according to many different parameters, and these properties play an essential role in determining the applications of nanofibers.^40^ Material selection plays a vital role in the mechanical properties of the produced nanofibers.^41^ The change of mechanical properties in the corneal stroma can lead to severe problems such as vision loss.^42^ Tensile strength and strain at break measurements were evaluated for the produced nanofiber patches and are shown in Figure 4. The tensile strength of the PVA/GEL nanofiber patch was found to be 8.86 ± 0.42 MPa, and strain at break was 26.9 ± 4.3%. When CA, GEN, and both drugs were loaded into PVA/GEL nanofiber patches, their tensile strengths were 7.96 ± 0.1, 7.35 ± 0.8, and 7.74 ± 0.9 MPa, respectively. The strain at break values were 18.32 ± 0.7, 5.74 ± 0.6, and 4.39 ± 0.2%, respectively. Loading of CA and GEN into PVA/GEL nanofiber patches caused a decrease in their tensile strength and strain at break values. Huang et al. investigated that when the hydrophilic drug GEN was loaded into the nanofiber patches, it was observed that the mechanical properties were decreased.^43^ In addition, as the drug was loaded into PVA/GEL nanofiber patches, fiber diameter was increased, as indicated in Figure 2, and mechanical properties were decreased with drug loading.

### 3.5 Swelling and Degradation Behavior of Nanofiber Patches

**Figure 5:**
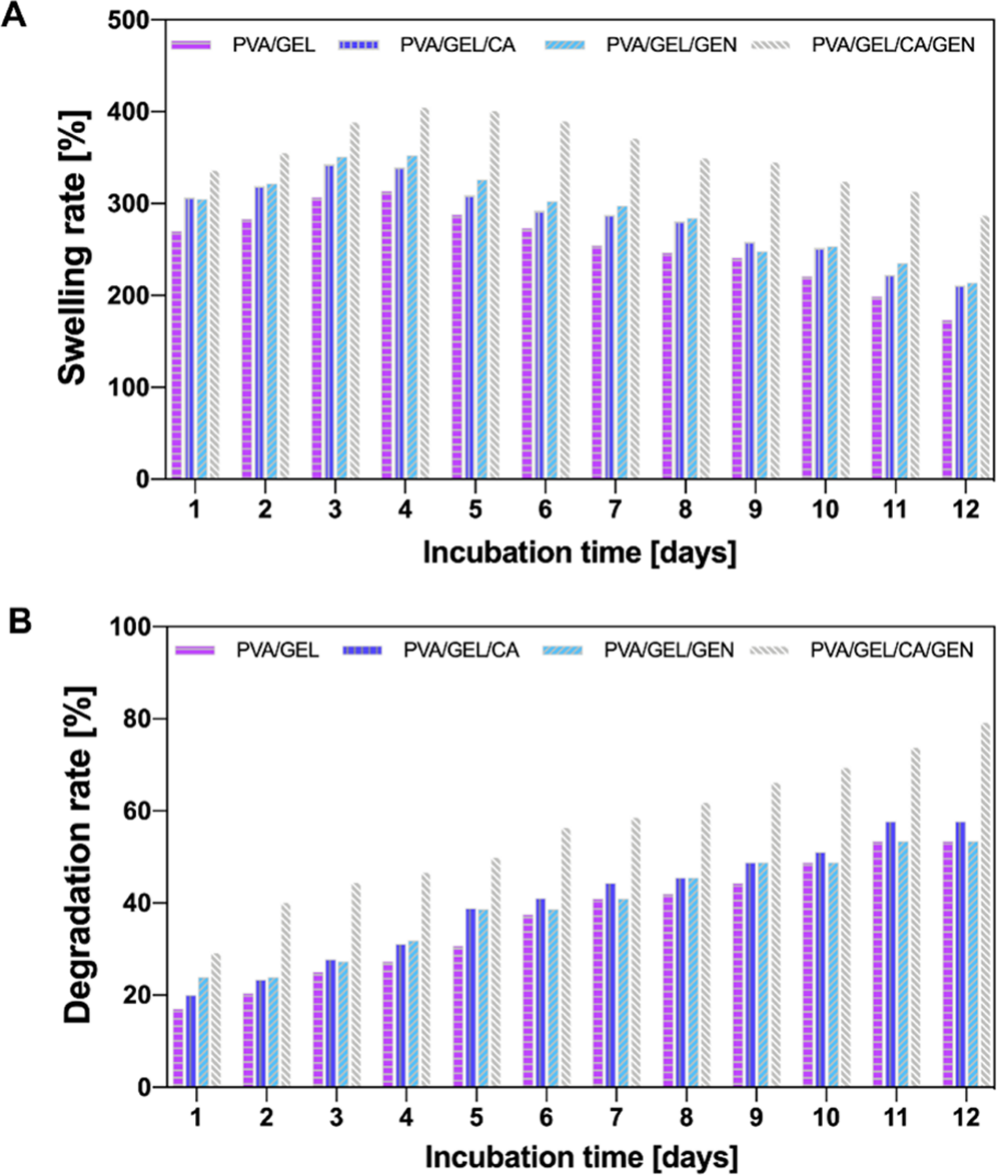
Swelling (A) and degradation (B) behaviors of nanofiber patches.

Swelling behavior is an important feature for determining structural stability and cell attachment of nanofiber patches.^44^ Swelling rates of the produced nanofiber patches at pH 7.4 and 37 °C are shown in Figure 5a. In the pure PVA/GEL nanofiber, the swelling rate, which continues until the end of the 4th day, increases to approximately 300%. After the 4th day, a degradation profile was observed instead of a swelling profile. For drug-loaded PVA/GEL/CA and PVA/GEL/GEN nanofiber patches, swelling behavior was observed by the end of 4th day. CA and GEN drug-loaded nanofiber patches started to show a degradation profile after the 4th day, and it was observed that the PVA/GEL/CA and PVA/GEL/GEN nanofiber patches showed similar swelling profiles with the pure group. It was also observed that PVA/GEL/CA/GEN nanofiber patch loaded with both drugs showed a higher swelling rate than other groups by approximately 400%. Although PVA/GEL/CA/GEN nanofiber patch exhibited a swelling profile until the end of the 5th day, it was noticeable that it started to degrade after 5 days, similar to the other nanofiber groups. Aydin et al. demonstrated that an increase in fiber diameter may cause an increase in swelling behavior.^45^ The higher swelling ratio of the PVA/GEL/CA/GEN nanofiber patches compared to the other groups may be associated with the higher diameter of the fibers. Structures exposed to PBS deteriorate over time lose their weight as they begin to dissolve in PBS.^21^ According to Figure 5b, degradation continued for 12 days. The degradation rate showed an increase for all nanofiber patch groups produced. PVA/GEL/CA/GEN nanofiber patch loaded with both drugs exhibited a higher degradation rate than other groups, of approximately 80%. Law et al. stated that with increasing drug loading, a decrease in tensile strength occurs because nanofibers provide water absorption and degradation due to their hydrophilic character.^46^

### 3.6 In Vitro Drug Release Test

In this study, the release of CA and GEN from nanofiber patches was performed. To mimic physiological conditions, all releases were performed in PBS at pH 7.4 and on a 37 °C shaker. For both agents, initially, solutions containing different drug concentrations were prepared (0.2, 0.4, 0.6, 0.8, and 1 μg/mL), and calibration curves were created. While the calibration curve was drawn according to the peak value obtained at 280 nm for CA, the peak curve was obtained for GEN at 196 nm (Figure S1A,C). Absorbance graphs were drawn for CA (*R*^2^ = 0.8968) and GEN (*R*^2^ = 0.9969), and correlation coefficients were obtained (Figure S1B,C).

**Figure 6:**
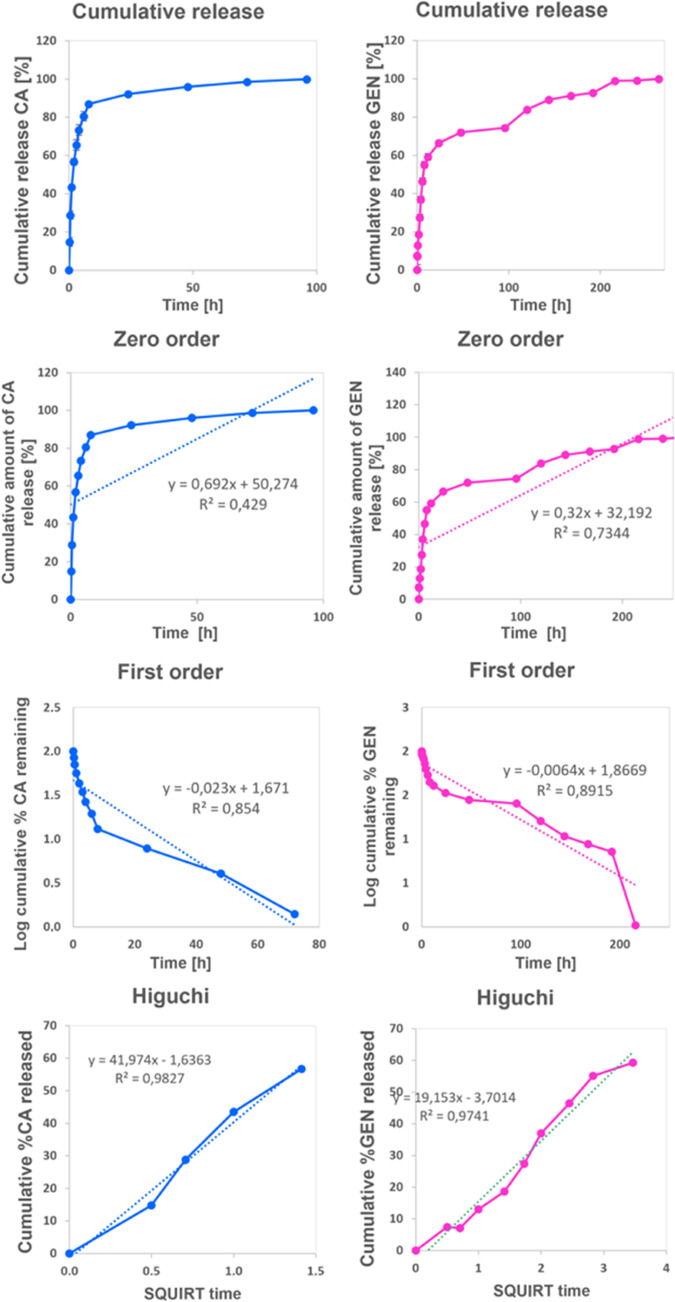

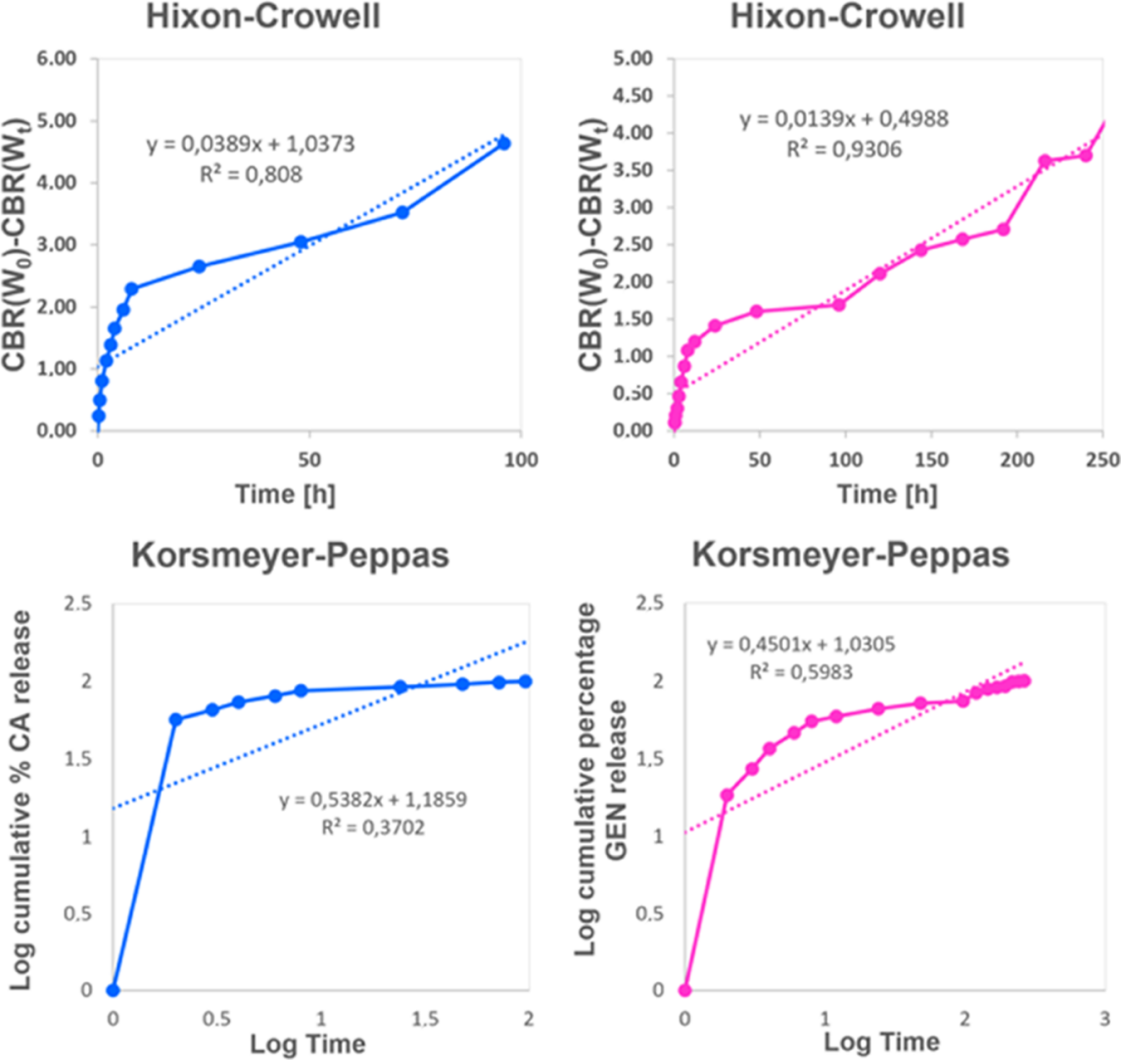
Zero-order, first-order, Korsmeyer–Peppas, Higuchi, and Hixson–Crowell kinetic models calculated for PVA/GEL/CA and PVA/GEL/GEN nanofiber patches.

According to the obtained cumulative release data, CA, showed burst release in the first 8 h with 87% and reached 100% after 96 h (Figure 6). CA (1.35 g L^–1^) is polar and has very low water solubility, and this might be the reason for the early release of the vast majority of the drug.^47^ Studies in the literature showed that all of the drug was released within the first hour when they examined the drug release from the CA-loaded PVA/Gillan nanofibers. The rapid release was explained by the hydrophobic nature of CA.^48^ When GEN release was investigated, the burst release occurred up to the 12th h, and 60.65% of the drug was released (Figure 6). However, it can be noticed that the release continues until the 264th h, and it can be seen that it drew a controlled release profile. The release of hydrophobic drugs like cinnamaldehyde from a carrier material made of polyvinyl alcohol and gelatin can be influenced by several factors, including the nature of drug-carrier interaction, the size and the shape of the drug molecules, and the properties of the carrier matrix. PVA is a hydrophilic polymer, and hydrophobic drugs like CA can form weak noncovalent interactions such as van der Waals, hydrophobic, and π–π stacking interactions with the PVA matrix. These interactions are weak and reversible and depend on factors like the surface area and molecular shape of the drug molecule. During the initial phase of drug release, the hydrophobic drug molecules present at or near the surface of the PVA matrix are quickly released due to the weak interaction between the drug and the carrier. This initial burst release can be attributed to the fact that the amount of drug released during this phase is generally high, and as the drug molecules move deeper into the carrier matrix, they experience stronger interactions with the carrier material, leading to a slower release rate. However, the release of gentamicin from PVA/Gel can be different from that of CA because GEN is a hydrophilic drug that can form hydrogen bonds with the hydrophilic PVA matrix. This can result in stronger drug-carrier interactions, leading to slower release rates compared to CA. Moreover, the size and shape of the drug molecule can also influence its release rate. For instance, a smaller drug molecule like CA may diffuse more quickly through the carrier matrix, leading to a faster release rate, while a larger drug molecule like GEN may be more challenging to release and may have a slower release rate.

The release kinetics of nanofiber patches were investigated with zero-order, first-order, Korsmeyer–Peppas, Higuchi, and Hixon–Crowell models. The kinetic constants and regression coefficients (*R*^2^) of nanofiber patches are given in Table 2. In nanofiber patches, the highest correlation number is associated with the appropriate mathematical model. The highest regression coefficient (*R*^2^ = 0.983) of the CA-loaded nanofiber patch was obtained in the Higuchi release model; similarly, the highest regression coefficient (*R*^2^ = 0.974) for GEN-loaded nanofiber was obtained for the same model (Figure 6). In addition, the ‘*n*’ values describing the drug release mechanism from the polymeric material correlated to the Korsmeyer–Peppas model (Table 3)^49^ presented in Table 3 suggest that the release follows the Super Case II mechanism of both drugs. Super Case II represents the mechanism of hydrophilic polymer swelling in liquid due to relaxation of its chains.^50^

**Table 2:** Correlation Coefficient *R*^2^ from In Vitro Release Data of CA and GEN for Different Release Kinetics Models

	regression coefficient *R*^2^ value					
nanofiber patch	zero-order	1st-order	Higuchi	Hixon–Crowell	Korsmeyer–Peppas	diffusion exponent (*n*)
PVA/GEL/CA	0.429	0.854	0.983	0.808	0.370	0.54
PVA/GEL/GEN	0.734	0.892	0.974	0.931	0.598	0.45

**Table 3:** Transport Mechanisms Types According to the Ranges of *n* Value

the ranges of *n* values	transport mechanisms
0.45 ≤ *n*	Fickian diffusion mechanism
0.45 < *n* < 0.89	Non-Fickian transport
*n* = 0.89	case II (relaxational) transport
*n* > 0.89	super case II transport

### 3.7 In Vitro Antibacterial and AntiBiofilm Activities of PVA/GEL/CA, PVA/GEL/GEN, and PVA/GEL/CA/GEN Nanofiber Patches

**Figure 7:**
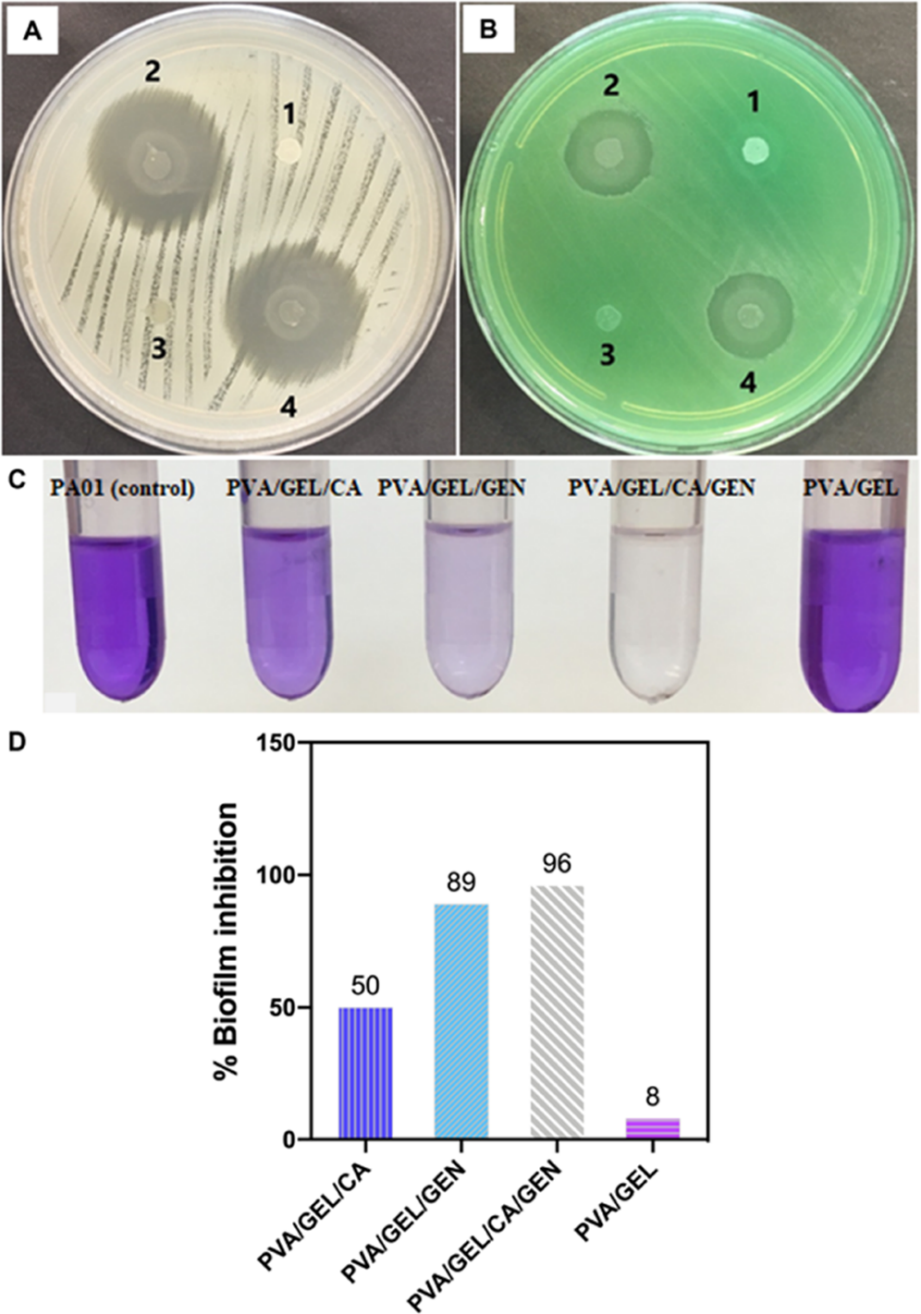
Antibacterial activity of PVA/GEL, PVA/GEL/GEN, PVA/GEL/CA, and PVA/GEL/CA/GEN nanofiber patches against *S. aureus* (A) and *P. aeruginosa* (B). (1) PVA/GEL, (2) PVA/GEL/GEN, (3) PVA/GEL/CA, and (4) PVA/GEL/CA/GEN nanofiber patches; (C) crystal violet (CV) staining of *P. aeruginosa* PA01 biofilm treated with PVA/GEL/CA, PVA/GEL/GEN, PVA/GEL/CA/GEN, and PVA/GEL nanofiber patches. More intense color reflects larger biofilm mass, and (D) percentage of biofilm inhibition by PVA/GEL/CA, PVA/GEL/GEN, PVA/GEL/CA/GEN, and PVA/GEL nanofiber patches assessed by CV quantification assay. Data are the means of three independent experiments.

The antibacterial activity of GEL/PVA nanofiber patches loaded with GEN or/and CA was evaluated. Disk diffusion test showed that PVA/GEL/GEN and PVA/ GEL/GEN/CA nanofiber patches have antibacterial properties against both *S. aureus* and *P. aeruginosa* (Figure 7). The results of the antibacterial activity of the nanofiber patches are shown in Table 4. As predicted, CA alone (PVA/GEL/CA) did not show antibacterial activity at the tested concentration against any of the tested bacteria. However, when CA was combined with GEN (PVA/GEL/GEN/CA), its presence enhanced the antibacterial effect of gentamicin (Figure 7). PVA/GEL/GEN/CA combination nanofiber patches exhibited better antibacterial activity (28 mm) than PVA/ GEL/GEN alone (26 mm) for *P. aeruginosa*. Similar results were obtained for *S. aureus*. While PVA/GEL/GEN nanofiber patches caused a 16 mm inhibition zone, a 17 mm inhibition zone was observed for PVA/GEL/GEN/CA. The visible outer circle surrounding the patches containing drugs (Figure 7A,B) results from the dissolution of the patches in the agar over time. Figure S2 shows the results of the disk diffusion tests of the drugs containing PVA/GEL/GEN/CA patches in the dry conditions, with prior drying of the agar surface in the incubator. The existing literature indicates the synergistic interaction of CA in combination with gentamicin against *P. aeruginosa* and *S. aureus*, which supports the presented findings.^51,52^ Biofilm formation in *P. aeruginosa* has been regarded as a crucial virulence feature that contributes to resistance against antimicrobial agents. In the present study, the biofilm formation of the *P. aeruginosa* PA01 strain in the presence of PVA/GEL/CA, PVA/GEL/GEN, and PVA/GEL/GEN/CA nanofiber patches was evaluated. PVA/GEL/CA and PVA/GEL/GEN nanofiber patches inhibited biofilm formation in *P. aeruginosa* by 50 and 89%, respectively, compared to the untreated *P. aeruginosa* PA01 (control) (Figure 7). Moreover, biofilm formation was abolished when CA was combined with GEN (96%) (Figure 7).

**Table 4:** Antibacterial Activity of PVA/GEL/CA, PVA/GEL/GEN, and PVA/GEL/GEN/CA Nanofiber Patches

	diameter of zone of inhibition (in mm)			
	PVA/GEL	PVA/GEL/CA	PVA/GEL/GEN	PVA/GEL/CA/GEN
*S. aureus*			16	17
*P. aeruginosa*			26	28

### 3.8 Cell Culture Test

#### 3.8.1 Cell Viability

**Figure 8:**
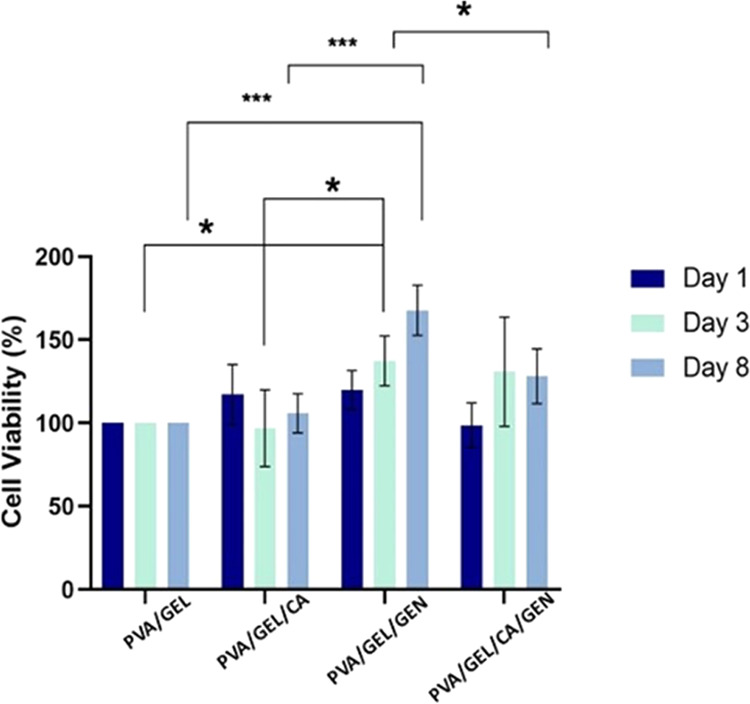
Percentage of cell viability within 8 days of in vitro culture with MEF cells. Dark blue: 1-day cell incubation with nanofiber, light blue: 3 days of nanofiber and cell incubation, and light gray: 8 days of cell and nanofiber incubation. The error bars indicate the standard deviation of 3 biological replicates of 3 technical replications. The PVA/GEL was used as the control group due to the raw material. *p < 0.05, ***p < 0.0001.

Cellular viability was calculated by comparing samples with the control nanofiber patch (PVA/GEL), and the comparison was applied for each period. The results showed that PVA/GEL/GEN exhibited higher cell viability compared with the control and all of the other samples after 8-day incubation (Figure 8). Also, cell viability of the cells cultured on PVA/GEL/GEN was significantly higher than observed for cells cultured on PVA/GEL and PVA/GEL/CA after 3 days of incubation. Additionally, the only statistical difference between the incubation time with PVA/GEL/GEN was from day 1 to day 8. Moreover, all three samples consisting of drugs had better cell viability than the PVA/GEL. Finally, the MTT assay results indicated that the materials had no toxic effects on the MEF cells.

#### 3.8.2 Fluorescence Imaging

**Figure 9:**
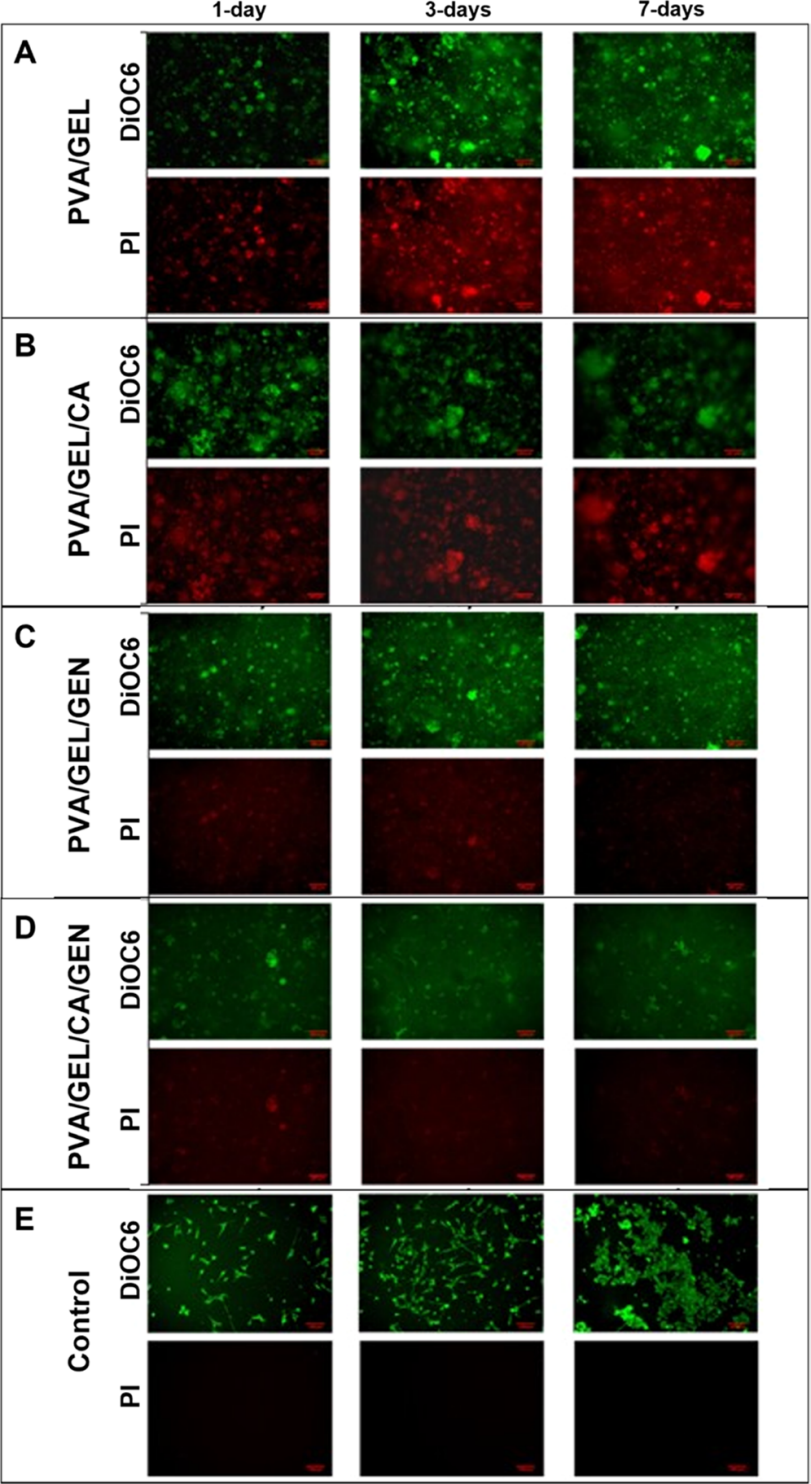
Fluorescence images for (A) PVA/GEL, (B) PVA/GEL/CA, (C) PVA/GEL/GEN, (D) PVA/GEL/CA/GEN, and (E) control (cells). Images were taken with ZOE Fluorescent Cell Imager, with 4 nM DiOC6 (green) and 0.5 mg/mL PI (red) staining. The scale bar is 100 μm.

**Figure 10:**
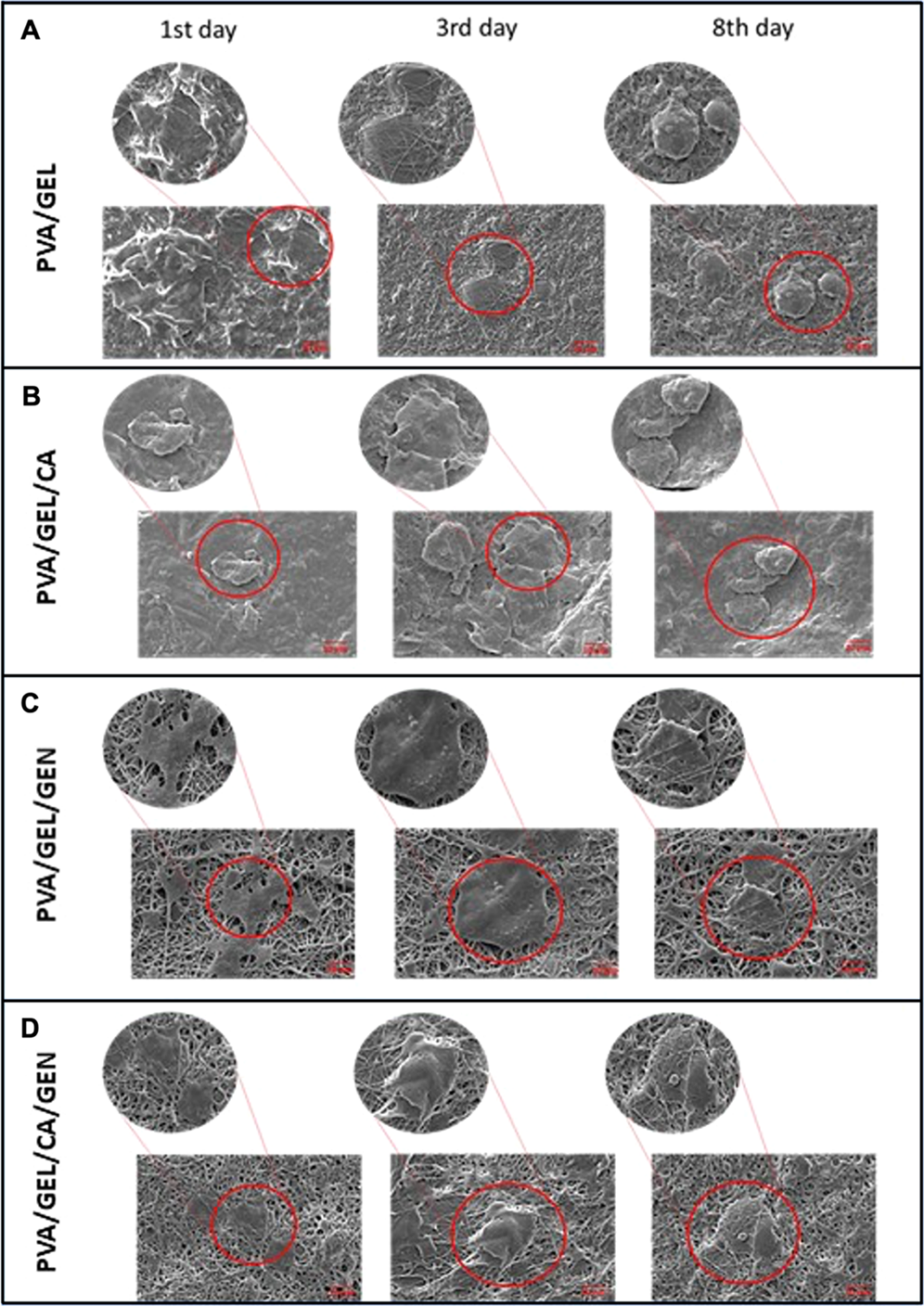
SEM images of the cells incubated with (A) PVA/GEL, (B) PVA/GEL/CA, (C) PVA/GEL/GEN, and (D) PVA/GEL/CA/GEN samples. The zoom-in images shown in the PVA/GEL have the scale of 2 μm on 1st day zoomed-in image. The other images have a 10 μm scale. The images have three columns indicating the three different time periods.

Fluorescence images were taken using DiOC6 and PI staining, and the results are presented in Figure 10. Identification of the cells on the nanofiber patch was challenging due to the cellular penetration into the material (Figure 9). The control group in the fluorescence images indicated the 2d cellular culture without samples (Figure 9E), and the morphology of the cells seemed accurate. However, the cellular morphology was not able to be distinguished when the cells were incubated with nanofiber patches.

#### 3.8.3 Morphology of the Cells Cultured on the Nanofibers

SEM images are shown in Figure 10, and the scale of the images was the same 10 μm, and the zoomed images were taken using a 2 μm scale. The PVA/GEL nanofiber patch had less fibril structure than the other nanofiber patches (PVA/GEL/GEN and PVA/GEL/CA/GEN), and the brighter areas visible on the SEM images might be attributed to the lower cellular attachment. Additionally, the cells had a round-shape, with longer incubation time. PVA/GEL patch was not toxic but less biocompatible compared to PVA/GEL/GEN and PVA/GEL/CA/GEN. The images taken for PVA/GEL/CA had less fibril; besides, the cellular exocytotic materials were observed on the surface of the cells for all three-incubation times. Even though the cellular viability increased compared with the PVA/GEL, which was shown by the MTT assay (Figure 8), the cellular morphology was similar for cells cultured on both materials (Figure 10, PVA/GEL/CA 3rd column).

Cells cultured on PVA/GEL/GEN had the best morphology and exhibited the best capability of attachment. Cellular attachment started after the first day of incubation. Moreover, the exosomes were observed on the fibril structure of the PVA/GEL/GEN nanofiber patch, which means that the cells communicate with each other. Finally, cellular attachment, viability, and signaling were observed for all incubation periods without any exceptions.

The cells cultured on PVA/GEL/CA/GEN nanofiber patch were attached to the surface, and the cellular morphology was observed as usual; however, the extracellular vesicles were observed on the cell with the zoomed image for first day of incubation. Cellular attachment and the exocytosis of intracellular exosomes were observed for PVA/GEL/CA/GEN nanofiber patch with 3-day cellular incubation; moreover, after 8-day cell incubation with PVA/GEL/CA/GEN, the cellular extension was enhanced, and the cells were combined with the nanofiber patches surface.

Although the biocompatibilities of these four samples after cellular experiments were accurate, PVA/GEL/GEN nanofiber patch supported cell viability, showed great cellular attachment, and provided cellular communication.

## 4 Conclusions

In this study, two-layer poly(vinyl alcohol)/gelatin (PVA/GEL) nanofiber patches containing CA in the first layer suppressing the biofilm formation and GEN with antimicrobial effect in the second layer were produced by the electrospinning method. SEM images showed that drug-free and drug-loaded nanofiber patches with bead-free and smooth surfaces were obtained, and the nanofiber size increased as the amount of drug increased. The tensile test results showed that the mechanical strength decreased as the drug was loaded. According to the swelling and degradation behavior results, it has been observed that especially the PVA/GEL/CA/GEN nanofiber patches have a very high water absorption capacity, and simultaneously they exhibit faster degradation. According to the drug release results, while CA release ended at the 96th hour, GEN release continued until the 264th hour. The successful release of CA and GEN was further confirmed by the antibacterial tests. The results of the disk diffusion test demonstrated that CA- and GEN-loaded PVA/GEL nanofiber patches exhibited significant antibacterial activities against *P. aeruginosa* and *S. aureus*. Similarly, an excellent biofilm inhibition in *P. aeruginosa* was observed in the presence of PVA/GEL/GEN and PVA/GEL/CA/GEN nanofiber patches. Additionally, these nanofibers have no cytotoxicity to human cells. PVA/GEL/GEN is the most promising material for cell survival, with high biocompatibility properties. Collectively, these results demonstrated that PVA/GEL/GEN and PVA/GEL/CA/GEN nanofiber patches might increase the success of antibiotic treatment by inhibiting biofilm formation and can be developed as an alternative strategy in the treatment of corneal infections such as bacterial keratitis.

## Supporting Information Available

Absorption spectra for CA and GEN and calibration curves for CA and GEN (PDF)
