# Correction to “Design of Cinnamaldehyde- and Gentamicin-Loaded Double-Layer Corneal Nanofiber Patches with Antibiofilm and Antimicrobial Effects”

**DOI:** 10.1021/acsomega.4c03133

**Published:** 2024-06-03

**Authors:** Sumeyye Cesur, Elif Ilhan, Tufan Arslan Tut, Elif Kaya, Basak Dalbayrak, Gulgun Bosgelmez Tinaz, Elif Damla Arısan, Oguzhan Gunduz, Ewa Kijeńska-Gawrońska

Upon careful review, it has come to our attention that Figure 2 in the originally published article has been incorrectly placed and contains inaccuracies. Figure 2 in question was inadvertently published in our article despite containing some SEM images and histograms of similar material being a previously published article titled “The Role of Multilayer Electrospun Poly(Vinyl Alcohol)/Gelatin nanofibers loaded with Fluconazole and Cinnamaldehyde in the Potential Treatment of Fungal Keratitis” (10.1016/j.eurpolymj.2022.111390).

We are issuing a correction to make adjustments to Figure 2 in this paper primarily involving the representation of the fibers and their respective diameters within the text. The revised images closely resemble the original ones, and the altered fiber diameters remain within the same range as the initial measurements. Given the nature of these changes, it is unlikely that they significantly affect the main findings of the paper or alter any values in the results that would impact the conclusions drawn. The core insights and conclusions of the study remain robust, as the adjustments made are confined to visual elements and do not introduce substantial variations in the underlying data or methodologies employed. We apologize for the oversight and the inconvenience it may have caused.

The diameters in section 3.1 and Figure 2 should be corrected as follows:

**3.1. Morphology of the Nanofibrous Patches.** (···) As shown in Figure 2, PVA/GEL, PVA/GEL/GEN, PVA/GEL/CA, and PVA/GEL/CA/GEN nanofiber patches were produced with mean diameters of 226 ± 49, 235 ± 24, 287 ± 75, and 322 ± 34 nm, respectively. (···)

**Figure 2:**
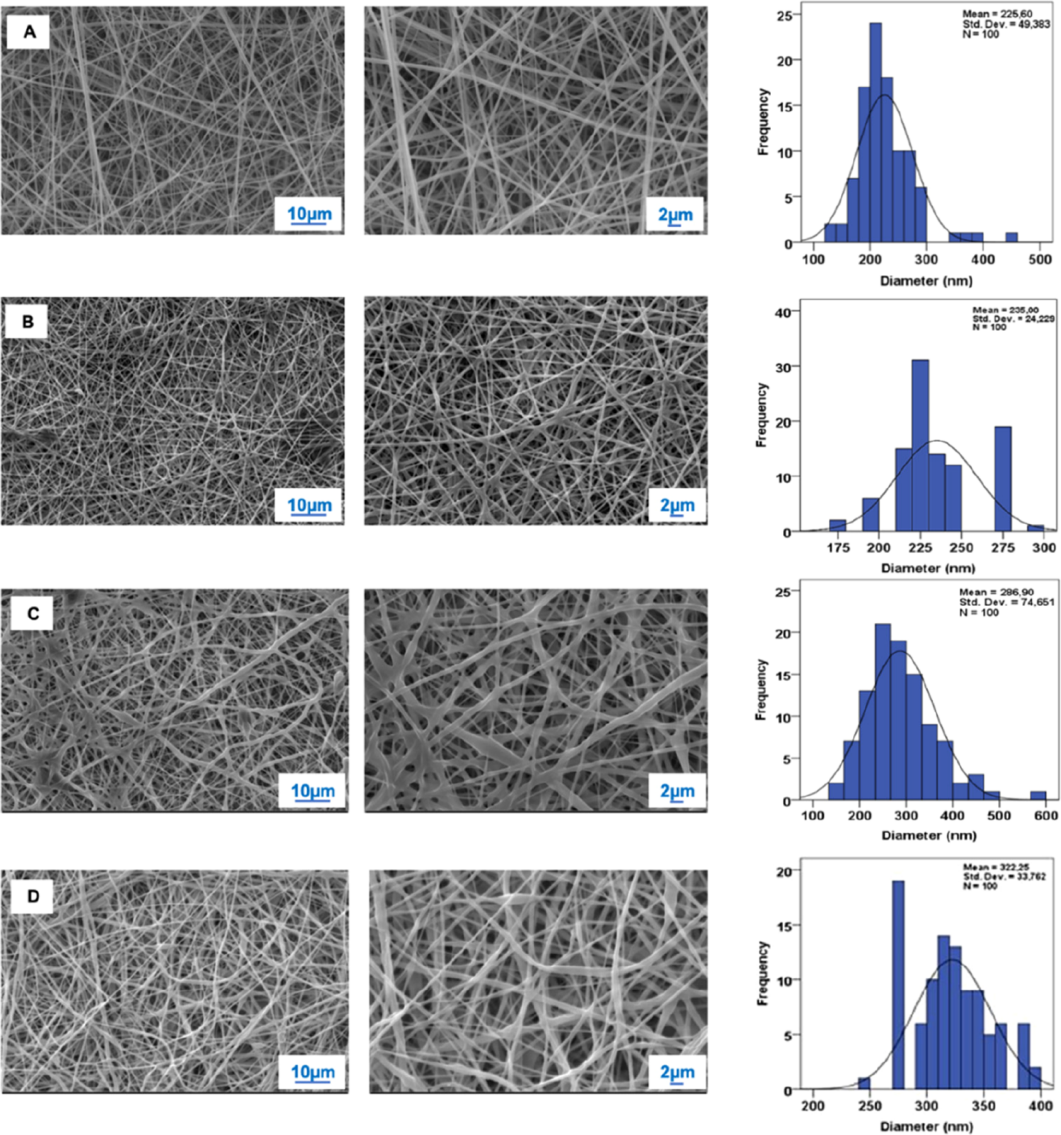
SEM images of the (A) PVA/GEL, (B) PVA/GEL/GEN, (C) PVA/GEL/CA, and (D) PVA/GEL/CA/GEN nanofiber patches and their fiber diameter distributions.

