# Supplementary material for: Design of Cinnamaldehyde- and Gentamicin-Loaded Double-Layer Corneal Nanofiber Patches with Antibiofilm and Antimicrobial Effects
Source: ACS Omega. 2023 Jul 26;8(31):28109–21. doi: 10.1021/acsomega.3c00914 (PMC10413367; doi:10.1021/acsomega.3c00914)
Supplement: Supplementary file 1 — ao3c00914_si_001.pdf [file ao3c00914_si_001.pdf]

Supplementary data

Design of Cinnamaldehyde and Gentamicin Loaded Double-Layer Corneal Nanofiber Patches with Antibiofilm and Antimicrobial Effects

Sumeyye Cesur[a,b], Elif Ilhan[a,c], Tufan Arslan Tut[a,b], Elif Kaya[d], Basak Dalbayrak[e], Gulgun Bosgelmez Tinaz[d], Elif Damla Arısan[e], Oguzhan Gunduz[a,b], Ewa Kijeńska-Gawrońska[f,g,*]

[a]Center for Nanotechnology & Biomaterials Application and Research (NBUAM), Marmara University, Turkey

[b]Department of Metallurgical and Materials Engineering, Faculty of Technology, Marmara University, Turkey

[c]Department of Bioengineering, Faculty of Engineering, Marmara University, Turkey

[d]Department of Basic Pharmaceutical Sciences, Faculty of Pharmacy, Marmara University, Istanbul, 34668, Turkey

[e]Department of Biotechnology, Institute of Biotechnology, Gebze Technical University, Gebze, Kocaeli, Turkey

[f]Centre for Advanced Materials and Technologies CEZAMAT, Warsaw University of Technology, Poland

[g]Faculty of Materials Science and Engineering, Warsaw University of Technology

* ewa.kijenska@pw.edu.pl

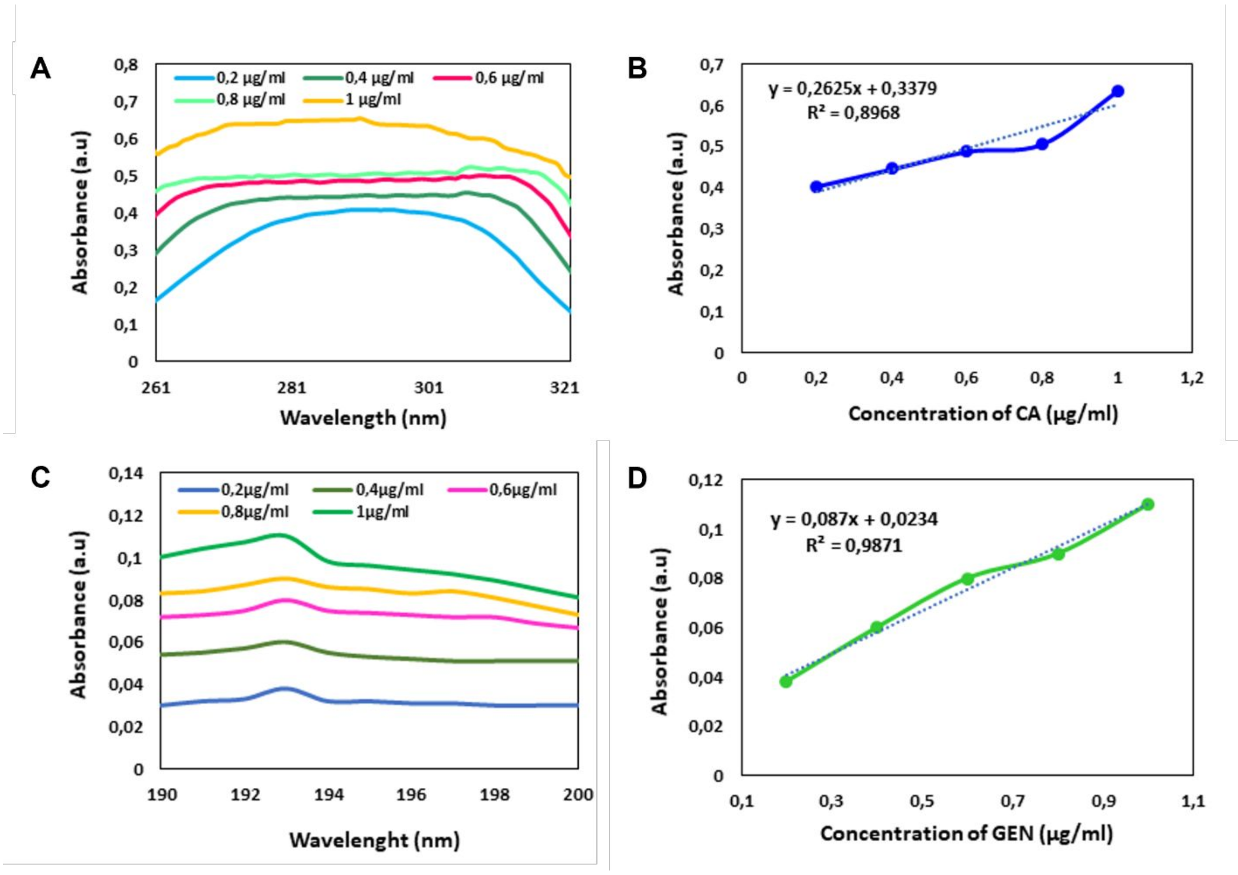

Figure S1. Absorption spectra of CA at different concentrations (A), CA calibration curve (B), absorption spectra of GEN at different concentrations (C), and GEN calibration curve (D).

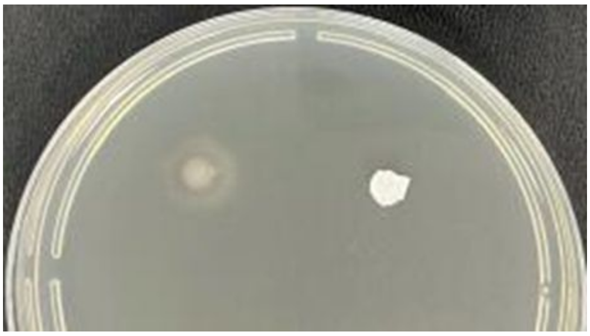

Figure S2. Optical image of the patch placed on the agar under dry conditions

Figure S2 shows the results of the disc diffusion tests of the drugs containing PVA/GEL/GEN/CA patches in the dry conditions, with prior drying of the agar surface in the incubator. The reason for the outer circle around the patches observed during ZOI experiments results from the dissolution of the patches in the agar over time.
